# Estimating spatially distributed turbulent heat fluxes from high-resolution thermal imagery acquired with a UAV system

**DOI:** 10.1080/01431161.2017.1280202

**Published:** 2017-01-31

**Authors:** Claire Brenner, Christina Elisabeth Thiem, Hans-Dieter Wizemann, Matthias Bernhardt, Karsten Schulz

**Affiliations:** ^a^ Institute of Water Management, Hydrology and Hydraulic Engineering, University of Natural Resources and Life Sciences, Vienna, Austria; ^b^ Institute of Physics and Meteorology, University of Hohenheim, Stuttgart, Germany

## Abstract

In this study, high-resolution thermal imagery acquired with a small unmanned aerial vehicle (UAV) is used to map evapotranspiration (ET) at a grassland site in Luxembourg. The land surface temperature (LST) information from the thermal imagery is the key input to a one-source and two-source energy balance model. While the one-source model treats the surface as a single uniform layer, the two-source model partitions the surface temperature and fluxes into soil and vegetation components. It thus explicitly accounts for the different contributions of both components to surface temperature as well as turbulent flux exchange with the atmosphere. Contrary to the two-source model, the one-source model requires an empirical adjustment parameter in order to account for the effect of the two components. Turbulent heat flux estimates of both modelling approaches are compared to eddy covariance (EC) measurements using the high-resolution input imagery UAVs provide. In this comparison, the effect of different methods for energy balance closure of the EC data on the agreement between modelled and measured fluxes is also analysed. Additionally, the sensitivity of the one-source model to the derivation of the empirical adjustment parameter is tested. Due to the very dry and hot conditions during the experiment, pronounced thermal patterns developed over the grassland site. These patterns result in spatially variable turbulent heat fluxes. The model comparison indicates that both models are able to derive ET estimates that compare well with EC measurements under these conditions. However, the two-source model, with a more complex treatment of the energy and surface temperature partitioning between the soil and vegetation, outperformed the simpler one-source model in estimating sensible and latent heat fluxes. This is consistent with findings from prior studies. For the one-source model, a time-variant expression of the adjustment parameter (to account for the difference between aerodynamic and radiometric temperature) that depends on the surface-to-air temperature gradient yielded the best agreement with EC measurements. This study showed that the applied UAV system equipped with a dual-camera set-up allows for the acquisition of thermal imagery with high spatial and temporal resolution that illustrates the small-scale heterogeneity of thermal surface properties. The UAV-based thermal imagery therefore provides the means for analysing patterns of LST and other surface properties with a high level of detail that cannot be obtained by traditional remote sensing methods.

## 1. Introduction

The amount of evapotranspiration (ET) along with its variability in space and time is important to quantify for effectively managing water resources in agricultural systems (Jiang and Islam 2001; Cleugh et al. 2007; Allen et al. 1998; Anderson et al. 2011; Brutsaert 2005; Bastiaanssen et al. 2005). However, the direct measurement of ET at high temporal resolutions (i.e. 30 min) that can be provided by eddy covariance (EC), Bowen ratio (BR), or scintillometer systems is time consuming and expensive to operate and furthermore is limited to an integral signal within a footprint of a few 10 to hundreds of meters (Foken 2008a).

Spatially distributed estimates of ET are mostly based on exploiting land surface temperature (LST) information gained from thermal infrared remote (TIR) sensing mounted on satellite or airborne platforms. The current operational satellite platforms offer a compromise between spatial and temporal resolutions of TIR data ranging from 3 km/15 min for the Meteosat Second Generation; 1 km/daily for the MODIS; to 100 m/16 days for the Landsat 8 platform. When ET estimates at high spatial and temporal resolutions are required, only airborne platforms are able to provide the necessary data.

The use of unmanned aerial vehicles (UAVs) has recently gained increasing attention in the remote sensing community due to the low costs of the UAV platforms as well as camera systems in the visible, near-infrared, and thermal spectral range (Zhang and Kovacs 2012; Candiago et al. 2015; Link, Senner, and Claupein 2013; Lelong et al. 2008; Berni et al. 2009; Turner et al. 2014). However, the UAV-based acquisition of spatially distributed and multi-temporal LST data and subsequent derivation of turbulent land surface energy fluxes including a quality assessment against EC measured data are rare (Hoffmann et al. 2016) and require more detailed investigation.

The basic principle of deriving latent heat fluxes from LST is to first calculate the sensible heat flux which is driven by a temperature gradient between the land surface and the atmosphere and a corresponding resistance term. Once the sensible heat flux is known, the latent heat flux can be derived as the residual term in the energy balance equation (see Section 2 for details). Despite the apparent simplicity of this approach, it is complicated by the inequality of the aerodynamic temperature (governing the heat exchange between the surface and the overlying atmosphere that cannot be measured directly) and the radiometric temperature seen by the radiometer (Norman and Becker 1995). This inequality occurs mainly due to differences in the thermodynamic temperatures of the soil and vegetation. The soil and vegetation contribute to the radiometric temperature proportionally to their fraction occupied in the radiometers field of view. In contrast, they contribute to the aerodynamic temperature in proportion to their resistance to turbulent heat exchange with the near-surface atmosphere (Norman, Kustas, and Humes 1995).

While a large number of different approaches have been developed (see Gowda et al. (2008), Kalma, McVicar, and McCabe (2008), Li et al. (2009) for comprehensive reviews), two of the most prominent schemes for deriving ET from LST are presented here. One-source energy balance (OSEB) models treat the surface as big leaf and therefore as a single uniform layer. They yield a bulk transfer of turbulent heat exchange without distinction between different potential sources, such as the soil and canopy (Bastiaanssen et al. 1998; Su 2002). The OSEB models have empirical adjustment parameters to account for differences in the aerodynamic and radiometric. The derivation of this adjustment parameter has been discussed by several researchers (Boulet et al. 2012; Kalma and Jupp 1990; Massman 1999; Verhoef, DeBruin, and van den Hurk 1997; Colaizzi et al. 2004). In the literature, various different expressions of this adjustment parameter exist of which several are based on the so-called *kB* ^−1^ parameter which expresses the difference in resistance to heat and momentum transfer (Troufleau et al. 1997; Matsushima 2005; Boulet et al. 2012).

The second prominent model is the two-source energy balance (TSEB) model that addresses the problem of the ambiguous relationship between aerodynamic and radiometric temperature by partitioning turbulent energy fluxes and net radiation into a soil and a vegetation component. ET models based on this approach include the original TSEB model (Norman, Kustas, and Humes 1995) and models that adopt the principle idea of the TSEB model such as the DTD model (Norman et al. 2000) and ALEXI (Anderson et al. 2007). While the original TSEB model uses instantaneous LST measurements, the DTD and ALEXI model both estimate ET based on the time rate of change in LST during the morning hours. Hoffmann et al. (2016) applied the TSEB and DTD model to thermal images acquired with a fixed wing UAV over a barley field. They found that energy fluxes modelled by both two-source models were in good agreement with EC measurements with the DTD outperforming the original TSEB model. However, the time-integrated DTD model requires flights at two times during the morning hours, thus complicating flight planning. In their study, Hoffmann et al. (2016) showed that small UAVs provide data suitable for mapping ET at the field scale.

However, no comparative evaluation of the simpler OSEB models and the more complex TSEB models exists for the high-resolution imagery that UAVs provide. Several inter-comparison studies of OSEB and TSEB models driven by satellite LST imagery concluded that TSEB models generally outperform OSEB models, showing better agreement with EC tower-based measurements (Gonzalez-Dugo et al. 2009; Long and Singh 2012; Timmermans et al. 2007; Tang et al. 2013, 2011; Gao and Long 2008; Choi et al. 2009).

This study will compare turbulent energy flux estimates of OSEB and TSEB models on the basis of UAV imagery with high spatial and temporal resolution. We used an octocopter UAV with a dual-camera set-up to map ET at a grassland site in Luxembourg. The experiment took place in July 2015 which was a period with extremely dry and hot conditions. Firstly, we compare the performance of the TSEB and OSEB models in reproducing field scale energy fluxes. Secondly, we investigate the impact of different expressions of the radiometric kB^−1^ parameter (the adjustment parameter in OSEB models to account for the difference between aerodynamic and radiometric temperature) on energy flux estimates by the OSEB model. The EC measurements serve as a baseline to test the model performances. The proposed approach thereby produces an integral over the source area, similar to the EC method, and also provides spatially explicit patterns which reveal the small-scale variability and the diurnal cycle of turbulent energy fluxes.

## 2. Data and site description

### 2.1. Study site

**Figure 1.**
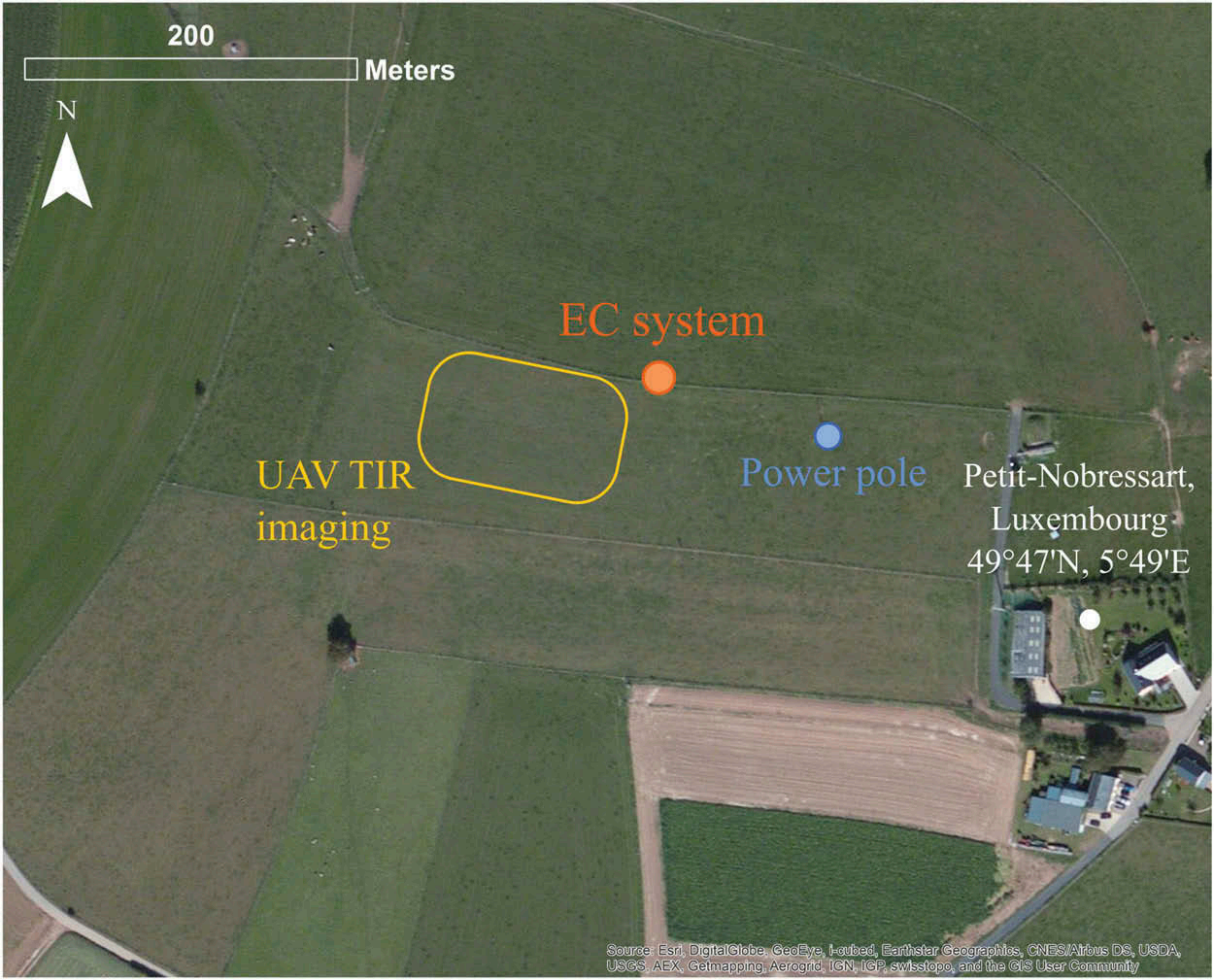
Overview of the study area and instrumentation. The study area is a grassland site in Petit-Nobressart, Luxembourg. Background: ESRI ® World Imagery.

**Figure 2.**
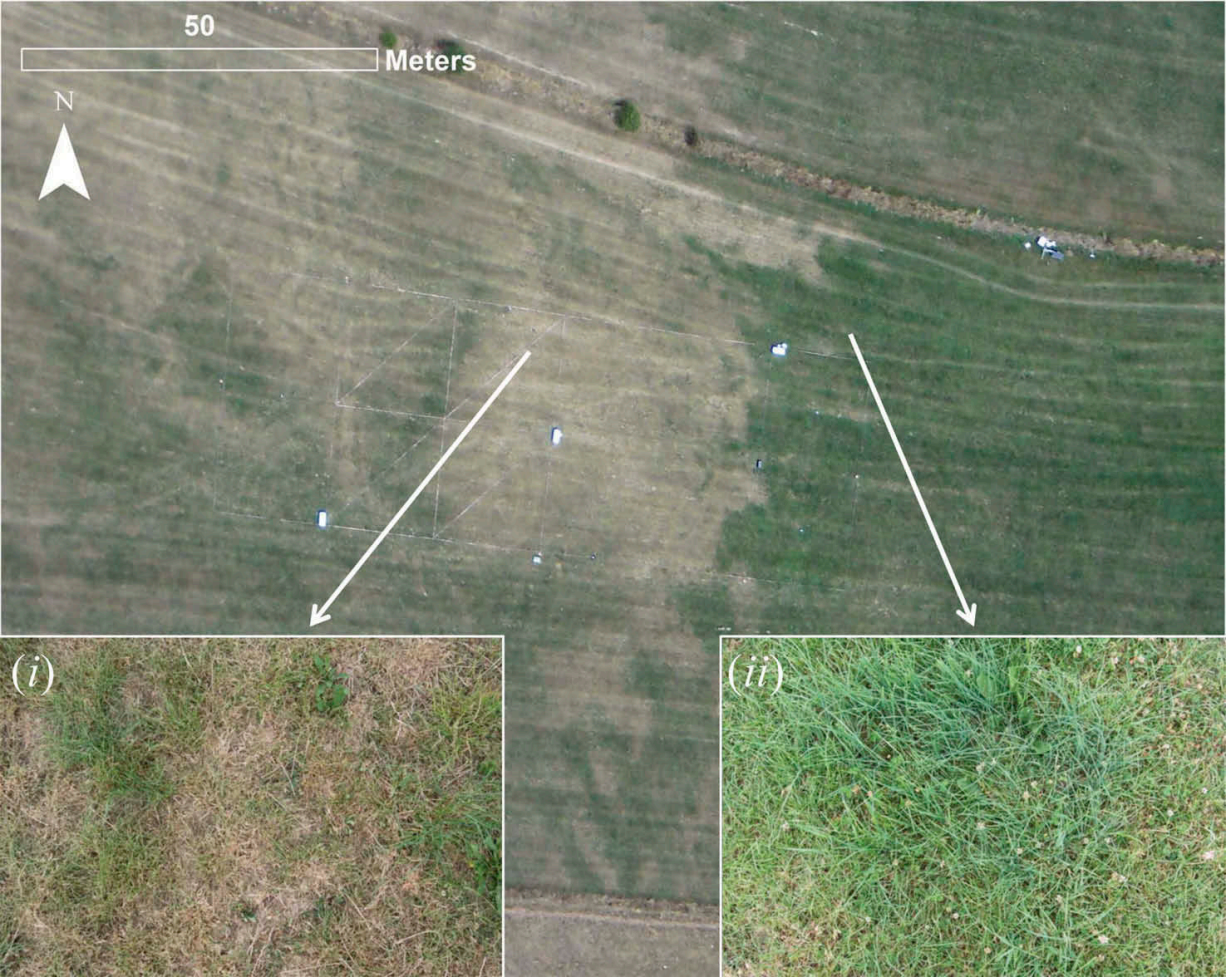
Overview of the state of the vegetation over the UAV footprint (see UAV TIR imaging area in Figure 1). The two inserts show examples of dried out vegetation (i) as it occurred mainly in the upper part of the slope (West) and more vital vegetation (ii) as it occurred mostly in the lower part of the field (East).

The study was conducted over a grassland site in Petit-Nobressart, Luxembourg, situated at a gentle east-facing slope. Figure 1 shows an overview of the experimental site and the instrumental set-up. The grassland was used as a hay meadow and had short vegetation of about 10–15 cm as the grass was mowed before the start of the experiment. The field campaign period in July 2015 was characterized by clear sky conditions with remarkably high air temperatures (daily maxima above 30°C) and no precipitation. Hence, the grass showed clear signs of water stress and turned brown especially in the upper part of the slope while it remained green and vital in the lower, flat part of the field (see Figure 2 (i,ii), respectively). The leaf area index (LAI) of the vegetation was assumed to range between 0.8 and 2 m^2^ m^−2^ based on close-up images of the vegetation. The normalized difference index using information from the red and green channels of a RGB image was used as a proxy for vegetation vitally (Pérez et al. 2000; Woebbecke et al. 1992). Based on this index, spatial patterns of LAI were generated by scaling between the set minimum and maximum LAI values.

Whenever thermal imagers or radiometers are used to measure surface temperatures, the thermal emissivity (*ε*) of this surface becomes a crucial parameter. Without knowing the thermal emissivity of a surface, no unique solution for the surface temperature from radiometer readings exists. This is due to the interrelationship between emissivity and surface temperature defined by the Stefan–Boltzmann law:

(1)
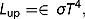

where *L*_up_ is the upwelling long-wave radiation, *σ* is the Stefan–Boltzmann constant, *ε* is the thermal emissivity of the surface and *T* is its thermodynamic temperature.

In this study, the thermal emissivity was set to a constant value of 0.98. This assumption is based on measurements of long-wave upwelling radiation and air temperature and an approach similar to Holmes et al. (2016). The basic idea is that the air and surface temperature are equal under conditions of zero sensible heat flux (around sunset and dawn). In these cases, the surface temperature can be set to the air temperature and the emissivity can then be estimated using radiometer measurements of upwelling long-wave radiation (see Equation (1)). Times with negligible sensible heat fluxes were identified using measurements from the EC system. Applying Equation (1) to these time steps yielded an emissivity value of 0.98, which is in accordance with Coll et al. (2001) and Rubio et al. (2003).

### 2.2. Micrometeorological data

An EC system consisting of a three-dimensional sonic anemometer (CSAT3, Campbell Scientific Inc.) and an open path infrared gas analyser (LI 7500, LI-COR Biosciences Inc.) was situated south of a grass strip separating two fields (see Figure 1). Besides the turbulent components, humidity, air temperature, precipitation, and the four radiation components (NR01, Hukseflux Thermal Sensors) were measured. Ground heat flux measurements with a set-up including soil heat flux plates, soil temperature, water content, and matric potential probes completed the instrumentation at the EC site. Turbulent fluxes were calculated using the EC software package TK 3.1(Mauder and Foken 2015). For the calculation of the ground heat flux, the flux values generated by the heat flux plates were corrected for heat storage in the soil layer above the plates.

#### 2.2.1. Footprint analysis

The footprint of the fluxes measured by the EC system describes the source area of the fluxes depending on wind direction, wind speed, and atmospheric stability. Knowing the footprint is essential for an accurate comparison of modelled and measured fluxes. In this study footprints were calculated using the forward Lagrangian stochastic trajectory model developed by Rannik et al. (2003) and Göckede et al. (2006).

Due to a power line crossing the field approximately at the flying altitude, the lower elevated southeast part of the field could not be covered by UAV flights. Therefore, only parts of the thermal imagery that were collected over areas with similar vegetation and thermal properties as the source area of the EC measurements were selected for the comparison.

#### 2.2.2. Energy balance closure of the EC data

The applied energy balance models close the surface energy balance by default. This means that the sum of ground heat flux, latent heat flux, and sensible heat flux equals the net radiation. However, this is not the case for EC measurements, where the sum of latent and sensible heat is systematically less than the available energy (net radiation minus ground heat flux) (Foken 2008b; Foken et al. 2010). For guaranteeing a fair comparison with the model results, fluxes measured by the EC station were adjusted in order to reach energy balance closure. Three different methods regarding the partitioning of the residual energy term were applied: in the BR method, the residual energy was added to both sensible and latent heat fluxes in order to preserve the BR (Twine et al. 2000). In both other cases, the whole residual energy was entirely added to either of the two fluxes, sensible heat or latent heat, respectively (Ingwersen et al. 2015).

### 2.3. UAV set-up and design of the flight campaign

UAV data were collected within the first week of July 2015. In total 16 flights were conducted at different times of day on DOY 181, 182, 183, 184, 187 as shown in Table 1. The time schedule was designed for covering the diurnal cycle of LST. An octocopter (MikroKopter OktoXL) with a payload of up to 4 kg was used as a platform and was equipped with a compact digital camera (Samsung ES80) and a thermal imager (Optris Pi 400), both mounted on a single brushless gimbal. The Optris Pi 400 with a weight of just 380 g is especially designed for aerial thermography. It detects thermal radiation in the spectral range from 7.5 to 13 μm and has a thermal sensitivity of 80 mK and accuracy of ±2.0°C. The optical resolution is 382 × 288 pixels with a field of view of 38° × 29° (f = 15 mm). At a flying altitude of 25 m, the ground resolution is around 5 cm. For all flights, the UAV flew along the same predefined waypoints with a ground speed of 2 m s^−1^ at a height of 25 m. For each flight, a series of images was collected at an 8-m interval and processed into an orthomosaic using Agisoft PhotoScan Professional.

**Table 1.** Overview of flight times for all UAV flights.

No.	Date (DOY)	**Time** (hh:mm)	No.	Date (DOY)	**Time** (hh:mm)
1	30 June (181)	10:58–11:08	9	3 July (184)	11:10–11:18
2	1 July (182)	11:08–11:16	10	3 July (184)	13:04–13:12
3	2 July (183)	06:17–06:25	11	3 July (184)	15:04–15:12
4	2 July (183)	09:03–09:11	12	6 July (187)	09:26–09:34
5	2 July (183)	11:01–11:09	13	6 July (187)	10:13–10:21
6	2 July (183)	13:01–13:09	14	6 July (187)	13:05–13:13
7	2 July (183)	18:16–18:24	15	6 July (187)	15:24–15:32
8	2 July (183)	21:00–21:08	16	6 July (187)	17:59–18:07

## 3. Methods

### 3.1. Orthomosaicing of the thermal imagery

Images acquired during the UAV flights were assembled to a single orthomosaic (orthorectified image) per flight for the thermal and optical imagery, respectively. Agisoft PhotoScan Profession that was used for orthomosaic generation is based on the structure from motion method (Westoby et al. 2012), a photogrammetric approach for building three-dimensional models of an object or topography from a series of overlapping photographs taken from different locations and orientations. Edges and local features that are visible in more than one image are used for image alignment. Compared to the optical imagery from the regular digital camera, the thermal imagery of the observed grassland site had less defined edges and smoother transitions. Thus, a workflow making use of the paired camera set-up (thermal imager and regular digital camera) was used for mosaicing of the thermal imagery. In a first step, the optical imagery was used to generate a three-dimensional model of the land surface. Since both cameras were mounted on the same gimbal with a known distance between the two focal points, information on camera locations and orientations derived from this work step could be used as input for the alignment and mosaicing of the thermal imagery. These data are much more accurate than positional estimates from the UAV’s GPS unit and thus facilitated proper image alignment. Based on this information on camera coordinates/orientations and the generated three-dimensional land surface model, all optical and thermal images were assembled into a single optical and thermal orthomosaic, respectively.

In general, information on camera calibration parameters including accurate values of the focal length, focal point position, and distortion parameters enhance the generation of accurate orthomosaics. Thus, the thermal imager was calibrated in the run-up to the field campaign using the freely available Camera Calibration Toolbox for Matlab ® (Bouguet 2016). The software that has originally been developed for RGB imagery needs multi-angle images of a calibration pattern, in this case a chequerboard pattern, as input. In order to enhance the contrast between the light and dark squares in the thermal spectrum, the white squares of the chequerboard were covered with aluminium foil. The difference in emissivities for the two materials intensifies the contrast between the squares. The software detects clearly identifiable features in the pattern (the corners of the squares of the chequerboard) and calculates the intrinsic camera parameters. The derived camera calibration values were input to the workflow in Agisoft PhotoScan Professional.

The two orthomosaics of LST and from the optical imagery, respectively, build the spatially distributed input for the ET modelling described in the following chapter.

### 3.2. OSEB – one-source energy balance model

In the used one-source model, evaporation is derived as the residual term of the surface energy balance equation (Equation (2)) after all other components are either measured or modelled.

(2)
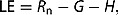

where LE is the latent heat flux, *R*_n_ is the net radiation, *G* is the ground heat flux, and *H* is the sensible heat flux. The ground heat flux is parameterized as a fraction of net radiation. The derivation of the sensible heat flux, *H*, is based on the difference between air and aerodynamic temperature and the aerodynamic resistance against this flux:

(3)
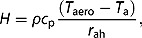

where *ρ* is the air density, *c*_p_ is the specific heat of air at constant pressure, *T*_a_ is the air temperature at a reference level, *T*_aero_ is the aerodynamic temperature, and *r*_ah_ is the aerodynamic resistance to heat transfer.

The aerodynamic temperature is defined as that temperature, which, when combined with the air temperature and a resistance calculated from the log-proﬁle theory, provides an estimate of the sensible heat ﬂux (Norman and Becker 1995). It occurs at an effective roughness height above the so-called zero displacement height. Aerodynamic temperature is unequal to the surface temperature measured by a radiometer especially over heterogeneous or sparse vegetation. The difference arises mainly from the effect of mixed pixels consisting partly of soil and canopy. While the soil temperature has a strong impact on the radiometric temperature (*T*_r_) observed by a thermal imager, its effect on the aerodynamic temperature is less pronounced (Boulet et al. 2012). During daytime, when the soil is typically warmer than the canopy, this results in the radiometric temperature exceeding the aerodynamic temperature by up to 10°C (Chehbouni et al. 1996). This unambiguous relationship between the two temperatures may lead to a significant overestimation of sensible heat fluxes. However, the aerodynamic temperature cannot be measured directly and thus has to be inferred from the measured radiometric temperature. The approach for relating these two temperatures is the main difference between one- and two-source models. While two-source models estimate separate soil and canopy temperatures from the mixed pixel information, one-source models introduce a parameter to adjust the radiometric to the aerodynamic temperature so that the measured radiometric temperature can be used in Equation (3) instead of *T*_aero_. This parameter is often expressed as function of the *kB* ^−1^ parameter that accounts for the difference in resistance to heat (*z*_oh_) and momentum transfer (*z*_om_) (Troufleau et al. 1997; Matsushima 2005). Thom (1972) found that the aerodynamic resistance to heat transfer is higher than to momentum transfer and results in a lower roughness length for heat than momentum exchange. The dimensionless aerodynamic *kB* ^−1^ parameter expresses this difference:

(4)
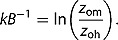

This ‘aerodynamic *kB* ^−1^’ is often assumed to be close to two (Garratt and Hicks 1973; Norman, Kustas, and Humes 1995; Kalma and Jupp 1990). However, in the literature the term *kB* ^−1^ was used confusingly for either this ‘aerodynamic *kB* ^−1^’ parameter or an adjusted ‘radiometric *kB* ^−1^’ parameter, that accounts next to the difference between the two roughness lengths also for the difference between aerodynamic and radiometric temperature (Matsushima 2005; Boulet et al. 2012). For clarity, these two *kB* ^−1^ values are distinguished by explicitly naming them ‘aerodynamic’ and ‘radiometric’ *kB* ^−1^. The following section discusses the derivation of an adjusted ‘radiometric *kB* ^−1^.’

The aerodynamic resistance to heat transfer, *r*_ah_, in Equation (3) is calculated based on the stability corrected log temperature and wind profile equations and can be expressed by:

(5)
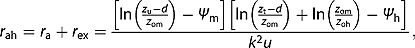

where *z*_u_ and *z*_t_ are the wind and air temperature measurement heights, respectively, *k* is the Karman constant, *d* is the zero displacement height, *u* is the wind speed, and *Ψ*_m_ and *Ψ*_h_ are the diabatic correction factors for momentum and heat (Brutsaert 2005). *r*_a_ is the aerodynamic resistance to momentum transfer and *r*_ex_ is an excess aerodynamic resistance arising from the higher resistance to heat transfer than moment transfer. *r*_ex_ is approximated by *kB* ^−1^/(ku*) with u* being the friction velocity (Garratt and Hicks 1973).

#### 3.2.1. Determination of the radiometric kB^−^^1^ parameter

Compared to the physically based aerodynamic *kB* ^−1^ value (see Equation (4)), the radiometric *kB* ^−1^ parameter is an empirical adjustment parameter for resolving the difference between the aerodynamic and radiometric temperature. Lhomme et al. (1997) and Troufleau et al. (1997) studied the behaviour of the *kB* ^−1^ parameter over sparse vegetation and found that it depends on vegetation properties, water availability, and climatic conditions and is thus not a constant parameter for a given vegetation type. However, several studies showed that over dense and homogeneous vegetation, energy fluxes modelled based on a constant *kB* ^−1^ value were in good agreement with measured fluxes (Kalma and Jupp 1990; Garratt and Hicks 1973).

In this study, three different expressions for *kB* ^−1^ were implemented in order to test the sensitivity of the model’s output to this parameter given the grassland cover at the experimental site:

- Constant *kB* ^−1^ value of 2.3
- Variable *kB* ^−1^ value according to Kustas et al. (1989)
- LAI dependent *kB* ^−1^ value according to Lhomme, Chehbouni, and Monteny (2000)

The most simplistic form is the use of a constant value for the *kB* ^−1^ parameter. For dense and homogeneous canopies, it can be assumed that there is little difference between the radiometric and aerodynamic temperatures. Thus, the ‘radiometric *kB* ^−1^’ component becomes less dominant. Therefore a value close to two seems appropriate for the *kB* ^−1^ parameter (Thom 1972; Kalma and Jupp 1990). In its earliest version the SEBAL model by Bastiaanssen et al. (1998) was parameterized with a fixed *kB* ^−1^ value of 2.3 that is also used in this study. Lhomme, Chehbouni, and Monteny (2000) established an empirical relationship between the LAI and *kB* ^−1^ in the form of a polynomial function of LAI and the radiometer zenith viewing angle. With the LAI ranging from 0.8 to 2 m^2^ m^−2^, the *kB* ^−1^ value according to this expression ranges from 2 to 4.9 over the experimental site. Kustas et al. (1989) defined *kB* ^−1^ as a function of the gradient between surface and air temperature as well as of wind speed

(6)
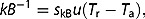

where *T*_r_ is the radiometric surface temperature, *T*_a_ is the air temperature, *u* is the wind speed, and *s*_kB_ is an empirical coefficient varying between 0.05 and 0.25. *s*_kB_ was set to 0.17 in accordance to Kustas et al. (1989). In the case of a negative temperature gradient (the surface is cooler than the air), this expression leads to a negative *kB* ^−1^ parameter. In the original paper by Kustas et al. (1989), Equation (6) was parameterized so that *kB* ^−1^ could not be less than zero. Also in this study, a minimum value of zero for *kB* ^−1^ was defined.

### 3.3. TSEB – two-source energy balance model

Compared to the one-source model, the TSEB model, developed by Norman, Kustas, and Humes (1995), partitions the surface temperature as well as surface fluxes into a soil and vegetation component. Detailed descriptions on the model can be found in Li et al. (2005) and Kustas and Norman (1999).

The surface energy budget is balanced for the soil and vegetation separately.

(7)
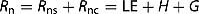

(8)
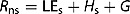

(9)
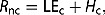

where the subscripts ‘s’ and ‘c’ represent the soil and vegetation/canopy component, respectively. Net radiation components are calculated based on measurements of incoming long- and short-wave radiation, vegetation and soil properties, and LST. Upwelling short-wave radiation is calculated separately for the vegetation and soil component based on reflectance properties of the both components. Upwelling long-wave radiation is also partitioned into a soil and vegetation component and calculated based on the component’s surface temperatures. Since the derivation of the component’s temperatures depends on measured radiometric temperature (*T*_r_), this measured composite temperature of the soil and canopy is the key input to the TSEB model:

(10)
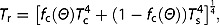

where *T*_s_ and *T*_c_ are the soil and canopy temperatures and *f*_c_(*Θ*) is the fractional vegetation cover at the radiometric field of view *Θ*. These component temperatures that are derived from the measured radiometric temperature drive the surface energy fluxes of the soil and canopy (see Equations (12)–(15)) and thus build the basis of the TSEB modelling strategy. However, given that Equation (10) is one equation with two unknowns, another equation for either of the two component’s temperatures is required. In the TSEB model, a first reasonable estimate of *T*_c_ is obtained by partitioning the net radiation in the canopy (*R*_nc_) into sensible and latent heat using the Priestley–Taylor equation (Priestley and Taylor 1972):

(11)
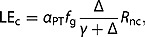

where *α*_PT_ is the Priestley–Taylor coefficient, *f*_g_ is the fraction of the vegetation that is green, *Δ* is the slope of the saturation water vapour–temperature curve, and *γ* is the psychrometric constant. As *H*_c_ is the residual between *R*_nc_ and LE_c_, *T*_c_ can be obtained from inverting Equation (12) which results from combining Equations (9) and (11).

(12)
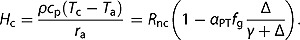

Based on this assumption, first estimates of the component temperatures and energy flux components can be obtained by solving the temperature gradient – resistances system of equations. In this study, sensible heat is calculated using a series resistance network (in contrast to a parallel resistance network that was used in the original model formulation) which accounts for the coupling of the soil and vegetation component by introducing an additional air temperature representative for the conditions within canopy stands *T*_AC_ (Kustas and Norman 1999; Timmermans et al. 2007; Colaizzi et al. 2012).

(13)
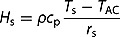

(14)
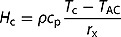

(15)
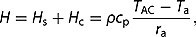

where *r*_s_, *r*_x_, and *r*_a_ are the resistances to momentum and heat transfer from the soil surface, canopy and atmospheric surface layer, respectively. Similar to OSEB models, the latent heat flux is estimated as the residual of the energy balance equation in this approach. However, starting with the assumption of potential transpiration assumed in the Priestley–Taylor equation, canopy latent heat fluxes may be overestimated at the expense of latent heat fluxes from the soil. In case that the vegetation is not transpiring at its potential rate, *T*_C_ may be underestimated (cooling effect due to evaporation is overestimated) and thus *T*_S_ and *H*_S_ are overestimated. This may lead to negative soil latent heat fluxes (LE_S_ < 0) during daytime (condensation at the soil surface) which is unlikely during daytime conditions. To avoid condensation at the soil surface, the α_PT_ coefficient is reduced incrementally until the soil latent heat flux becomes zero or positive. All other energy balance components are updated accordingly to satisfy the energy balance equation and meet the defined constraints. Consequently, a solution for the soil and canopy component is reached if soil latent heat fluxes are positive.

## 4. Results and discussion

### 4.1. Thermal imagery and vegetation patterns

**Figure 3.**
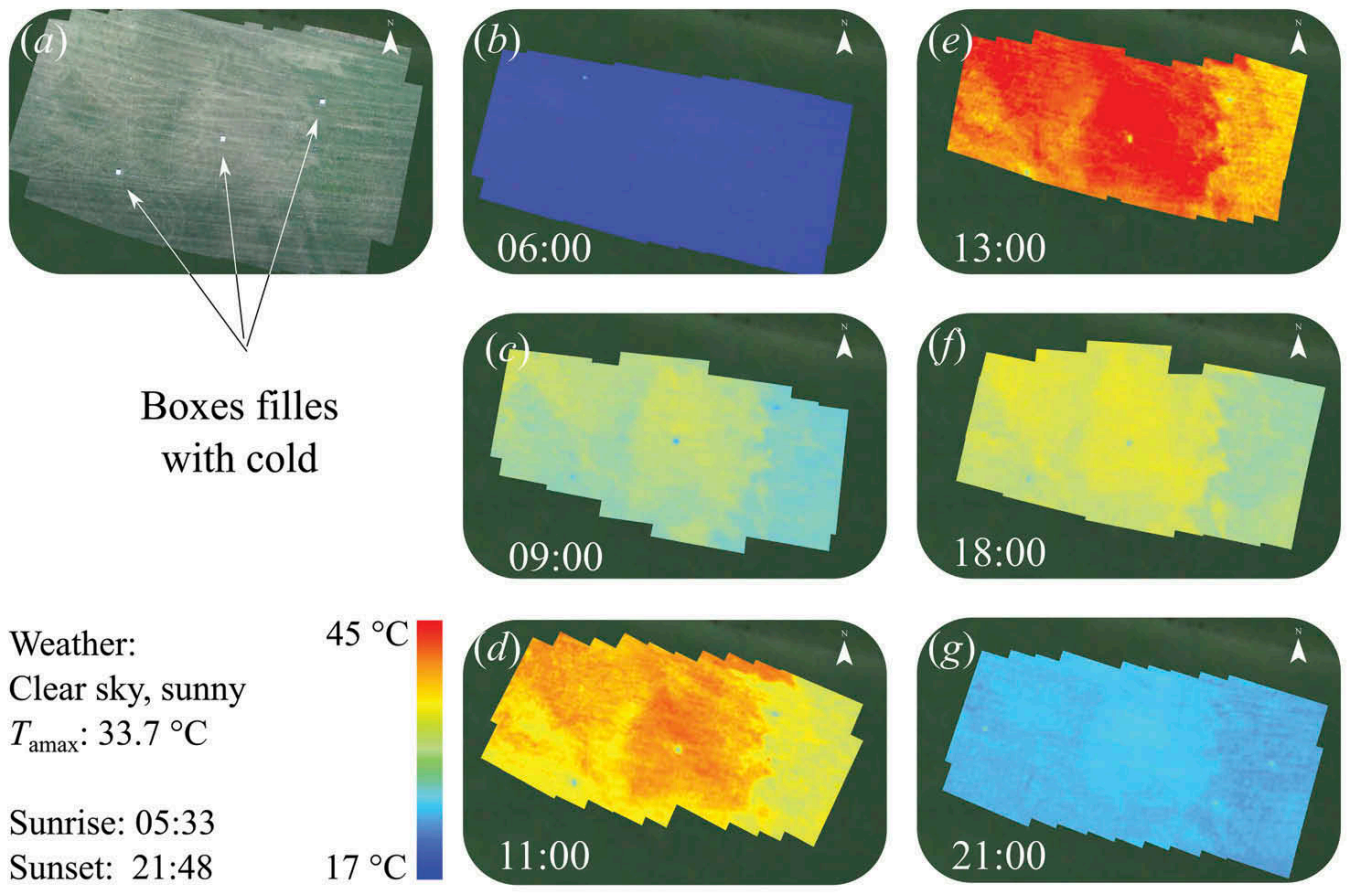
Example of optical and thermal imagery acquired on 2 July 2015 at six times of day (6, 9, 11, 13, 18, and 21 h). Times are in local time that is UTC + 02:00 (CET + daylight saving time). *T*_amax_ is the maximum half-hourly air temperature measured at the EC site.

Figure 3 shows an example of thermal imagery acquired on 2 July as well as a photograph of its source area. Three boxes filled with water that were used as reference points are visible on both the thermal and optical imagery. While the grass is still green in the western part of the field, it has turned brown in the centre part, creating a patchy vegetation structure. The same pattern is apparent in the thermal imagery, implying that the thermal signature is strongly linked to vegetation properties.

This small-scale heterogeneity is visible thanks to the high-resolution thermal imagery UAVs provide. In the case of coarser satellite imagery, the patchy vegetation structure and associated LST patterns vanish. However, the heterogeneous LST leads to strong small-scale variability in energy fluxes that can be assessed neither by field scale measurement techniques such as EC systems nor by coarser resolution imagery. Figure 3 not only shows the spatial heterogeneity but also the intensification of thermal patterns from morning to noon and its attenuation towards the evening. Especially in the data acquired around noon (Figure 3(d,e)), strong edges exist between cool and warm areas. The relatively homogeneous surface temperature over the area at 6 am (Figure 3(b)) supports the assumption of homogeneous emissivity values over the study area. Before sunrise, the spatial variability of LST is little since radiative forcing and turbulent exchange processes are low. Due to the nature of the relationship between emitted irradiance, emissivity, and temperature of a body (see Equation (1)), differences in emissivity for two surfaces would lead to differences in temperature readings by the radiometer even given that the two surfaces have the same actual surface temperature. As the thermal signal of the thermal camera is very homogeneous over the area, it can be assumed that the influence of spatially variable emissivity values is little.

### 4.2. Comparison of modelled and observed fluxes

**Figure 4.**
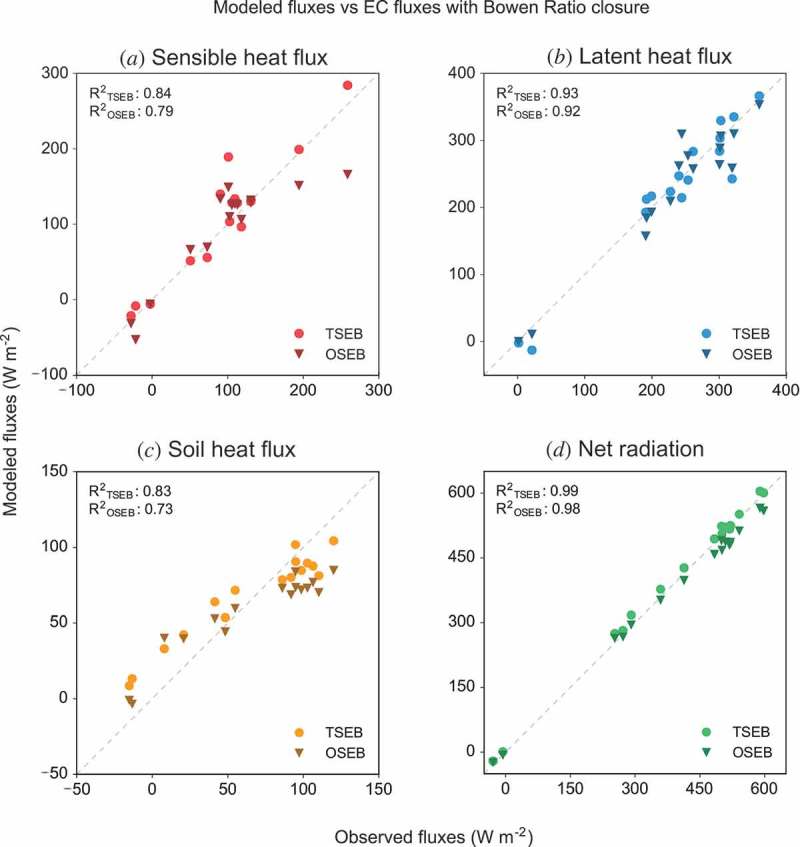
Comparison of modelled and measured energy balance components (latent and sensible heat, ground heat flux, and net radiation). Fluxes modelled by the OSEB model are marked with circles, while the TSEB output is marked with triangles. The 1:1 line is marked with a dashed line.

This section presents the comparison of the fluxes modelled by the one-source model (OSEB) and the two-source model (TSEB) with measurements from the EC system. For this comparison, the spatially distributed flux estimates from the models are averaged to a bulk flux over the source area, which is comparable to the EC measurement output. Figure 4 shows the comparison of modelling results and EC data for the turbulent fluxes as well as ground heat fluxes and net radiation. The EC data in Figure 4 are corrected for energy balance closure using the BR method (Twine et al. 2000), which yielded the best overall agreement between modelled and measured fluxes. For the OSEB model, it shows the results using the *kB* ^−1^ expression by Kustas et al. (1989) (see Equation (6)), which led to the best agreement with the EC measurements of latent heat. The effect of the three different *kB* ^−1^ estimation methods detailed above is discussed in Section 4.3.

**Figure 5.**
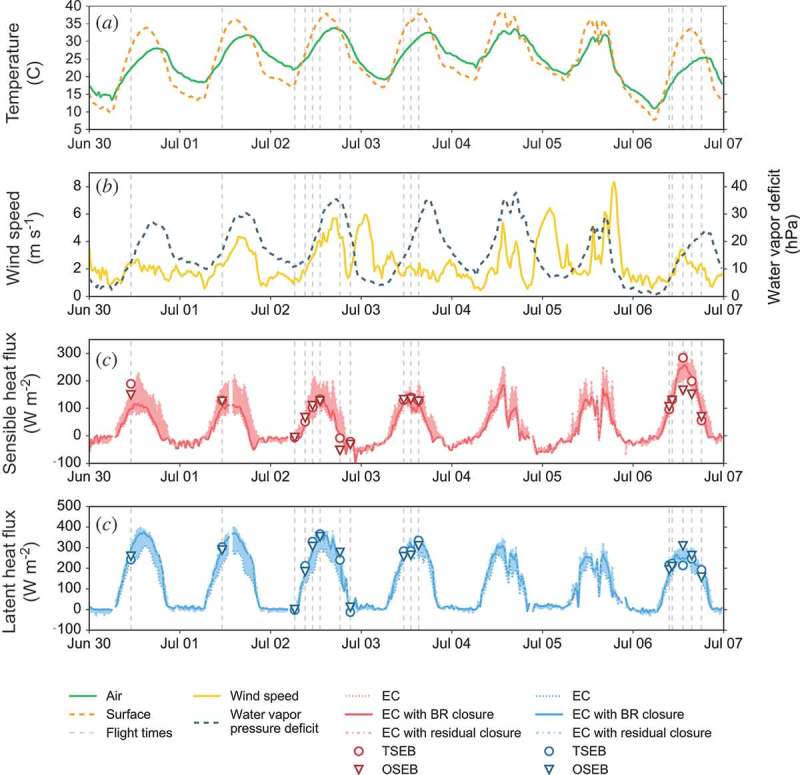
Time series of several meteorological variables and observed and modelled fluxes over the UAV field campaign period 30 June–6 July 2015. Depicted surface temperature was inverted from radiometer reading of upwelling long-wave radiation assuming the same value for emissivity (0.98) as for the UAV LST data. It is presented as proxies for the gradient between air and temperature over the entire field campaign. The vertical dashed grey lines represent times of UAV overflights. The shaded areas in (c) and (d) illustrate the difference between the uncorrected EC data and the data corrected by adding the entire residual term to either sensible or latent heat for the two plots, respectively.

Figure 5 gives an overview of meteorological conditions (air temperature, wind speed, water vapour deficit, radiation components) over the entire UAV field campaign. It also shows the EC-based time series of sensible and latent fluxes (with and without closure adjustments) as well as instantaneous fluxes modelled by the two energy balance models.

From Figure 4(b), it becomes clear that in general both models reproduce measured latent fluxes fairly well with R^2^ values of 0.93 and 0.92 for the TSEB and OSEB model, respectively. Concerning sensible heat fluxes, the OSEB model significantly underestimates measured values for high flux conditions. Interestingly, even though latent heat fluxes are calculated as the residuals of the energy balance equation after solving for the sensible heat component, both models performed better in reproducing latent heat than sensible heat fluxes. This can be explained by the inverse behaviour of the models in overestimating sensible and ground heat fluxes. The models tend to overestimate the sensible heat flux in cases where they underestimate the ground heat flux and vice versa. As these two deviations from the measured values compensate each other, latent heat fluxes estimated as residual terms in the energy balance equation (Equation (2)), are again in good agreement with the measured EC values. In general, as Figure 4(c) shows, both models reproduce ground heat fluxes with a reduced variance and systematically overestimate low and underestimate high ground heat fluxes. Both models calculate net radiation with negligible deviations from the measured values. Table 2 gives an overview of the statistics of model performances for the uncorrected EC data and all three closure-adjusted measurements.

**Table 2.** Difference statistics comparing model output of energy balance components from TSEB (TS) and OSEB (OS) and EC observations (raw observations and with adjustments for energy balance closure: Bowen ratio method (BR), residual LE method (LE res), residual H method (H res)) in W m^−2^.

Flux	Mean	Bias		MAE		RMSD		***R*^2^**	
	Obs	TS	OS	TS	OS	TS	OS	TS	OS
*R*_n_	395	−11	17	12	19	14	24	0.99	0.98
*G*	66	−2	9	16	20	18	23	0.83	0.73
*H*_raw_	74	−34	−19	35	30	46	36	0.34	0.61
*H*_BR_	95	−13	2	18	22	29	33	0.84	0.79
*H*_res_	152	43	58	50	61	60	77	0.45	0.13
LE_raw_	178	−52	−50	57	54	69	69	0.30	0.31
LE_BR_	234	4	6	18	20	26	28	0.93	0.92
LE_res_	255	25	27	28	33	39	39	0.86	0.86

Listed are the mean of the observations (Obs), the bias (Σ(O − M)/n), mean absolute error (MAE = Σ|O − M|/n), root mean squared difference (RMSD = [Σ(O − M)^2^/n]^1/2^), and coefficient of determination (*R* ^2^ = 1 − (Σ(O − M)^2^/Σ(O − 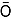)^2^), where n is the sample size, O is the observed, and M is the modelled value.

Figure 4(a) shows that the OSEB model significantly underestimates the measured sensible heat flux for two occasions with high fluxes (around 200 W m^−2^ and higher). Both data points with sensible heat fluxes above 200 W m^−2^ are from flights from the same day, 6 July, when the air-to-surface temperature gradient was especially high (see Figure 5(a)). The high vertical temperature gradient forces high sensible heat fluxes. However, in the case of the OSEB model, this gradient also affects the radiometric *kB* ^−1^ parameter, that accounts for the difference between aerodynamic and radiometric surface temperature. Especially for the data from the UAV flight at 13 h on 6 July, the calculated *kB* ^−1^ parameter is exceptionally high compared to all other flights with a value above eight (for flights around noon on other days it ranges between three to four). The higher this *kB* ^−1^ parameter, the higher is the resistance to heat transfer from the surface to the atmosphere. Thus, the high *kB* ^−1^ value forces low sensible heat fluxes and explains the poor performance of the OSEB model in this case compared to the TSEB model, which indeed was developed to circumvent the need for this empirical radiometric *kB* ^−1^ parameter.

In contrast, the TSEB model shows considerable deviations from the measured latent heat fluxes only for one time step. The corresponding LST data were collected on 30 June. In this case, the model underestimates the latent heat flux and overestimates the sensible heat flux. In principle, the OSEB model shows the same tendency. However, again in this case the very high temperature gradient between the surface and the air leads to a high *kB* ^−1^ value and thus reduced sensible heat fluxes.

### 4.3. Comparison of different expressions for kB^−1^ for the OSEB model

**Figure 6.**
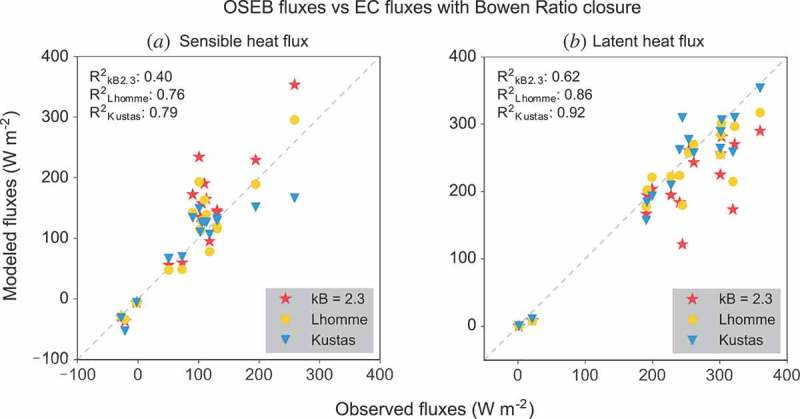
Comparison of modelled fluxes based on three different *kB* ^−1^ expressions. The 1:1 line is marked with a dashed line.

Homogeneous surfaces like full canopies are often assumed to act as isothermal surfaces for which the aerodynamic and radiometric surface temperature are identical. In this case, the ‘radiometric *kB* ^−1’^ parameter becomes superfluous. Even though grassland might generally be thought of as a homogenous, isothermal cover type, this assumption seems to be violated here due to the dry conditions and low vegetation density. As a consequence of the limited water availability, the grass dried out and became less dense whereby more of the soil became visible to the radiometer. This results in significant differences between aerodynamic and radiometric temperatures which in turn lead to overestimation of sensible heat fluxes assuming isothermal conditions. Figure 6 illustrates this case by assuming a fixed *kB* ^−1^ value of 2.3 (that represents mainly the aerodynamic part of the *kB* ^−1^ parameter assuming little difference between aerodynamic and radiometric temperature). Sensible heat fluxes are heavily overestimated in this case while latent heat fluxes are underestimated. The approach by Lhomme, Chehbouni, and Monteny (2000) calculates the *kB* ^−1^ value as a function of LAI. With the LAI values ranging between 0.8 and 2 m^2^ m^−2^, the *kB* ^−1^ value varies between 2 for the areas with denser vegetation cover and 4.9 for the areas where the vegetation is sparse. In general, this approach outperforms a fixed value, but systematically underestimates latent heat fluxes. The time-variant definition of *kB* ^−1^ proposed by Kustas et al. (1989), shows no such systematic behaviour but, depending on the meteorological conditions, might lead to over- or underestimation of the turbulent energy fluxes.

### 4.4. Sensitivity to surface temperature

The manufacturer of the thermal imager specifies an absolute accuracy of ±2.0°C. Tests prior to the field campaign showed that the accuracy of the thermal imager was well within the specified range with errors below ±1.0°C. During the flight, an automatic offset correction of the sensor done by a motor-driven motion of a blackened metal piece in front of the sensor prevents a thermal drift of the imager. However, to account for the uncertainty in absolute values, the sensitivity of both models to the absolute accuracy of LST values was analysed. For both models, turbulent heat fluxes were calculated with a modified input LST of ±2.0°C from the original value.

**Figure 7.**
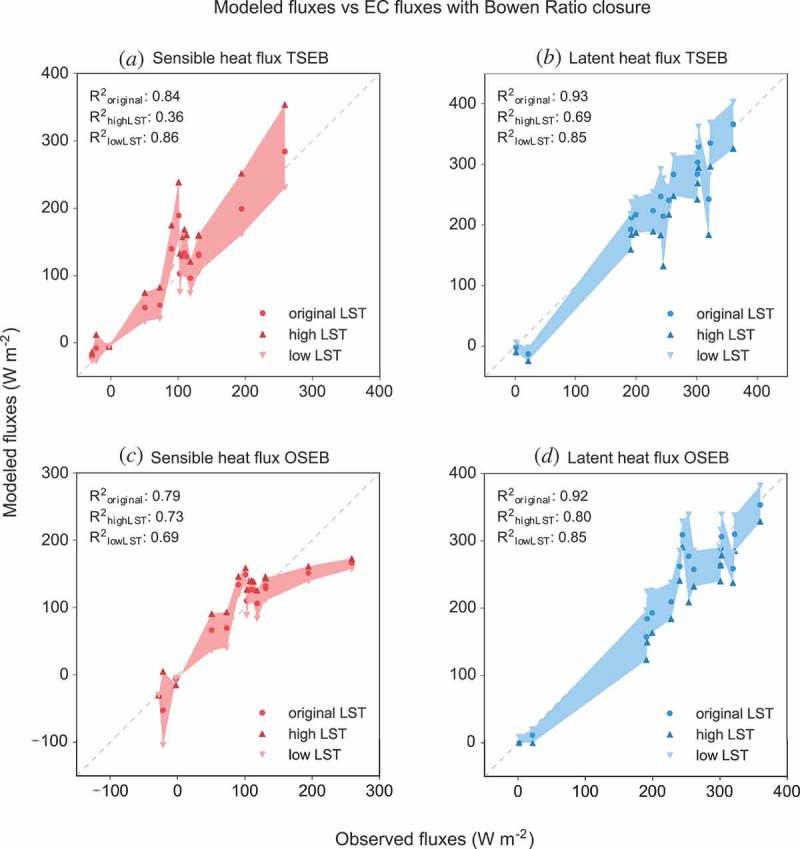
Turbulent heat fluxes of the TSEB and OSEB model with its original LST values, LST +2°C (high) and LST −2°C (low) temperature input. The shaded areas represent the ranges between flux estimates from model runs with increased (+2°C) and decreased (−2°C) surface temperature input. The 1:1 line is marked with a dashed line.

Figure 7 shows the results for the OSEB and TSEB models given the original, increased (−2°C) and decreased (+2°C) LST input. For both models, latent heat fluxes deviate stronger from EC measurements with increased LST values. Concerning sensible heat fluxes, fluxes calculated by the TSEB model deteriorate more for increased LST values, while the OSEB model shows the opposite behaviour being more sensitive to reduced LST values. However, in most cases the TSEB model has a higher sensitivity to temperature variation especially at high sensible heat fluxes. The lower sensitivity of the OSEB model to surface temperature variations can be traced back to the *kB* ^−1^ parameter, as expressed in Equation (6). The variation of LST affects the main driver of sensible heat flux, the temperature gradient between the surface, and the atmosphere. Higher gradients lead to higher sensible heat fluxes. However, the *kB* ^−1^ parameter increases as well with increasing temperature gradients and decreases in the opposite case. Since the resistance to heat transfer increases with high *kB* ^−1^ values, this parameter attenuates the effect of the temperature variations. Contrary, to its robustness against temperature variations under conditions with high sensible heat fluxes, the OSEB model is most sensitive to conditions with low sensible heat fluxes and thus low surface-to-air temperature gradients. For one data point in Figure 7(c) sensible heat estimates of the OSEB model vary between around zero and −100 W m^−2^. This data point belongs to a UAV flight at 18:00 on 2 July. As shows Figure 5(a), surface and air temperature are very close to each other for this point in time with the air temperature slightly exceeding the surface temperature. Under these conditions, the OSEB model parameterized with the *kB* ^−1^ parameter proposed by Kustas et al. (1989) becomes unstable. For both other occasions with low sensible heat fluxes (06:00 and 21:00, 2 July) the net radiation is low, so that the limited available energy prevents large absolute errors in flux estimates for these points in time.

## 5. Conclusion

In this study, we derive spatially distributed turbulent energy fluxes based on LST information acquired with a thermal imager mounted on an octocopter UAV. The high resolution of the imagery reveals small-scale variability of LST which results in variability in turbulent heat fluxes. A one- and a TSEB model were run using this high-resolution LST information to calculate sensible and latent heat exchange between the surface and the atmosphere. The two-source model accounts for the difference between the measured radiometric surface temperature and the aerodynamic temperature governing the heat exchange by partitioning surface temperature and fluxes into a soil and vegetation component. Contrary to the two-source energy model, the OSEB model needs an empirical adjustment parameter (*kB* ^−1^) to account for the difference between the radiometric and aerodynamic surface temperature. The sensitivity of the OSEB model to this parameter was tested by comparing flux estimates calculated with three different expressions of this adjustment parameter. Even though the TSEB model structure is more complex, the additional input requirements with more information needed on vegetation properties (e.g. LAI, fractional vegetation cover) are small.

The analysis showed that the spatially aggregated energy flux estimates of both models compare well to EC measurements, which inherently represent an integral flux over the source area. Regarding the EC measurements, the Bower Ration closure method yielded the best overall agreement between measured and modelled fluxes. However, for both sensible and latent heat fluxes, the more complex TSEB model outperformed the OSEB model with the difference being more pronounced for sensible heat flux. This is consistent with results from prior studies (Gonzalez-Dugo et al. 2009; Tang et al. 2013). The poorer performance of the OSEB model in reproducing sensible heat fluxes mainly results from an underestimation of high fluxes. This is mainly due to the kB^−1^ parameter which is overestimated in case of strong temperature gradients between the surface and the atmosphere. Of the three expressions for *kB* ^−1^ tested in this study, the time-variant parameterization by Kustas et al. (1989) led to flux estimates agreeing best to EC measurements. Both other expressions led to a systematic underestimation of latent heat fluxes and overestimation of sensible heat fluxes. A sensitivity analysis showed that the OSEB model is less sensitive to LST variations than the TSEB model. This reduced sensitivity is again related to the *kB* ^−1^ parameter that depends on the surface-to-air temperature gradient and thus varies likewise with the temperature gradient itself, which drives sensible heat fluxes. An increase in LST leads to an increased gradient between the surface and air temperature which results in higher sensible heat fluxes. However, this also increases the *kB* ^−1^ parameter which enhances the resistance against heat transfer and thus attenuates the effect of the higher gradient. In general, energy balance models depending on instantaneous LST information have a high sensitivity to uncertainties in the absolute value of this key input variable (Xia et al. 2015; Anderson et al. 1997; Norman et al. 2000). As discussed by Xia et al. (2015), a hybrid modelling concept integrating the more complex physically based TSEB model and a simpler contextual scaling scheme may enhance the overall robustness of ET estimates. These simpler contextual scaling approaches use temperature extremes of hot/dry and cool/wet pixels to scale fluxes over the area of investigation and are therefore less sensitive to the absolute value of LST. However, several inter-comparison studies showed that in general the TSEB model is more robust under a wide range of environmental conditions and derives flux estimates that are in better agreement with EC measurements (Xia et al. 2015; Timmermans et al. 2007; French, Hunsaker, and Thorp 2015; Choi et al. 2009). Thus, a combined approach of both model concepts may enhance the robustness of ET estimates.

UAVs are a rapidly developing technology that is increasingly used in agricultural management and water management practices. The UAV set-up applied in this study consisting of a thermal imager and a regular digital camera proved to be suitable for mapping spatially distributed turbulent heat fluxes at the field scale. The analysis demonstrated that thermal patterns, which result in heterogeneous turbulent heat flux patterns, are strongly linked to vegetation vitality. The analysis of patterns of LST along with other surface properties based on these high-resolution UAV data may contribute to a better understanding of the driving and limiting mechanisms of turbulent heat exchange processes in the future.
